# Simple and efficient HPLC-MS/MS method for simultaneous extraction and quantification of anthelmintics in equine manure

**DOI:** 10.1016/j.mex.2026.104042

**Published:** 2026-07-12

**Authors:** K. Holzer, U. Bertsche, M. Meyer, T. Schilling, J. Pfannstiel, L.E. Hoelzle

**Affiliations:** a University of Hohenheim, Institute of Animal Science, Dpt. Livestock Infectiology and Environmental Hygiene, Garbenstrasse 30, Stuttgart, 70599, Germany; b Core Facility Hohenheim, Mass Spectrometry Unit, University of Hohenheim, Ottilie-Zeller-Weg 2, Stuttgart, 70599, Germany

**Keywords:** HPLC-MS/MS, MRM, Anthelmintic, Pyrantel pamoate, Fenbendazole, Moxidectin, Ivermectin, Horse manure, Extraction

## Abstract

Anthelmintic treatment is a common practice in horses. The presence of pharmaceutical residues in fields and pastures is well-documented affecting various organisms, like nematodes.

This study describes the development and validation of a high-performance liquid chromatography tandem mass spectrometry (HPLC-MS/MS) method using multiple reaction monitoring (MRM) for extracting and determining the concentrations of four commonly used anthelmintics in horse manure. Manure was initially dried, then spiked with various concentrations (10–10,000 ng) of anthelmintics and extracted using an acetonitrile-water mixture containing 5 mM ammonium formate, with no need for further solid-phase extraction (SPE) cleaning. Recovery rates ranged from 82.3% for pyrantel to 120.8% for ivermectin, with standard deviations below 20%. Calibration curves were linear between 1 ng/mL and approximately 1.5 µg/mL for all four components. The lower limits of detection (LLODs) were 0.1 ng/mL for ivermectin, 0.2 ng/mL for moxidectin, 1.0 ng/mL for fenbendazole due to carryover, and 0.3 ng/mL for pyrantel, which formed two peaks.

• Novel method for simultaneous extraction of anthelmintics from horse manure.
• Method allows efficient and reliable detection of the mainly used anthelmintics in horses.
• Basis for the extension of the method to other applications, including the analysis of anthelmintics in soil samples.

## Graphical abstract

Created in BioRender. Hoelzle, L. (2026) https://BioRender.com/w9iu8m4.

**Figure:**
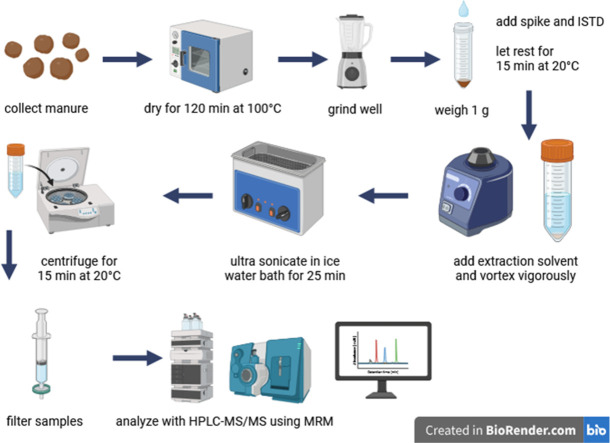

Specifications table.

**Table:** 

**Subject area**	Agricultural and Biological Sciences.
**More specific subject area**	Analysis of veterinary medicine.
**Name of your method**	Simultaneous extraction and analysis of four anthelmintics commonly used in veterinary medicine from equine manure.
**Name and reference of original method**	[1]
**Resource availability**	

## Background

Anthelmintics are widely used in veterinary medicine to control parasitic infections in livestock. These drugs play a crucial role in maintaining animal health and productivity. However, the widespread use of anthelmintics has raised concerns about their environmental impact, particularly when they are excreted in manure. Although the distribution of antiparasitics to veterinarians by industry and wholesalers is recorded by the federal states, much like antibiotics, this data is not systematically analyzed [2]. As a result, there is a lack of both distribution and usage data for these compounds. Horse manure, like other livestock waste, can serve as a significant source of environmental contamination. These drugs residues can persist in the environment, potentially affecting soil health, aquatic ecosystems, and non-target organisms, including beneficial insects and soil microorganisms [[3], [4], [5]]. The environmental behavior of anthelmintics has become a subject of growing research interest. One of the key challenges in studying the environmental impact of anthelmintics is the reliable extraction and quantification of these compounds from complex matrices like manure. To date, no straightforward extraction method has been developed for the simultaneous recovery of four commonly used anthelmintics from horse manure, namely fenbendazole, ivermectin, moxidectin, and pyrantel. Earlier methods for the extraction of moxidectin and ivermectin from horse manure involved solid phase extraction with several washing steps. The analytes were subsequently derivatized with N-methylimidazole, separated by high-performance liquid chromatography (HPLC) and detected by fluorescence [6,7]. The lower recovery rates of 72.4% for moxidectin with a standard deviation of 9.3% and 69.15% for ivermectin with a standard deviation of 9.01, respectively, may be attributable to the multistep sample preparation process [8]. An alternative approach to extracting fenbendazole from farm manure uses solid-phase extraction (SPE) prior to HPLC-MS/MS analysis [9].

The objective of this study was to develop a rapid and effective extraction method for the simultaneous isolation of commonly used anthelmintics for subsequent analysis by high-performance liquid chromatography-tandem mass spectrometry (HPLC-MS/MS). Here, we report the analysis of four anthelmintics belonging to distinct chemical classes from horse manure. Pyrantel pamoate is a pyrimidine derivative, fenbendazole is a benzimidazole, and moxidectin and ivermectin are macrocyclic lactones. This method could be used to gain insight into the environmental behavior of anthelmintics in general or the rate at which they degrade in the environment.

## Method details

### Chemicals

Fenbendazole-d_3_, moxidectin, pyrantel pamoate and dimethylsulfoxid (DMSO) were bought from Supelco (Darmstadt, Germany). Ivermectin, fenbendazole, and ammonium formate was from Sigma-Aldrich (Darmstadt, Germany); All solvents (formic acid, water, methanol, acetonitrile, and ethanol) were Hypersolv HPLC-MS grade from VWR (Darmstadt, Germany).

### Sample collection

Horse manure was collected from one horse that had not been treated with anthelmintics for at least three months. Horse manure was collected directly from the ground after defecation. The horse's age is unknown, but the average age of horses in this stable is seven years. The horse was fed hay or alfalfa haylage as its staple diet. When stabled overnight, the horse had access to straw bedding, which it could consume. Otherwise, the horse grazed on pasture. The horse had free access to salt licks and received oats, a specialized breeding muesli, and mineral supplements when stabled. The fecal samples were provided to us one day after collection. Upon receipt, the material was stored in the refrigerator for one to two days, then dried and homogenized using a blender. The dried fecal samples were stored at room temperature until extraction.

### Standard solutions, calibration curves and dilutions

A stock solution of ammonium formate was prepared at a concentration of 500 mM in water.

All anthelmintic stock solutions were prepared at a concentration of 1 mg/mL, with the requisite quantities of the respective substances weighed accordingly. The compounds fenbendazole and pyrantel pamoate were dissolved in dimethyl sulfoxide (DMSO), while the compounds ivermectin and moxidectin were dissolved in ethanol containing 5 mM ammonium formate. All solutions were stored at −70 °C and used for no longer than four weeks.

Spiking solutions of the four anthelmintics were frozen at −70 degrees and prepared freshly for each extraction round, containing 10 µg of each compound in 300 µL of a solution of DMSO and ethanol (47:52) with 5 mM ammonium formate. Subsequent spiking solutions were prepared through 10-fold dilutions extending down to 10 ng/300 µL.

Fenbendazole-d3 was utilized as the internal standard (ISTD), dissolved at a concentration of 1 mg/mL in DMSO and stored at −20 °C. Working solutions were prepared in DMSO and ethanol (47:52) and 5 mM ammonium formate at 4 µg/mL and stored at −70 °C.

Calibration curves were prepared by diluting 10 µL of the respective spike solutions and 20 µL of the ISTD into manure at a final volume of 333 µL. This resulted in concentrations of 1, 10, 100, and 1000 ng/mL. For 500 ng/mL, the spike solution was initially diluted at a ratio of 1:1.

When conducting the dilution experiments, the spike solutions and ISTD were directly diluted into the manure extract at the same ratio used during extraction.

### Sample preparation and extraction

The efficiency of the extraction method was evaluated by spiking defined concentrations of anthelmintics into dried horse manure. At first, horse manure was dried at 100 °C for 120 min in an incubator. Afterwards, 300 µL of the spiking solution were added to 1 g of dried manure to ensure an adequate ratio of spiking volume to manure. Subsequently, the spiked horse manure was combined with 500 µL of the internal standard (ISTD) solution. After a 15-minute incubation period at 20 °C, 3 mL of water, 5 mL of acetonitrile, and 80 µL of ammonium formate were added to the mixture. Ammonium formate was determined to be indispensable for the reproducibility and linearity of detection, particularly in the context of moxidectin and ivermectin, as had been previously reported [1]. The extraction solution was subjected to vigorous vortexing to ensure the complete homogenization of its components. The samples were subsequently subjected to ultrasonication in an ice-water bath for 25 min, to enhance the efficiency of the extraction process. The mixture was then centrifuged at 4.600 rpm (4020 g) for 15 min at 20 °C, to separate solid and liquid phases. The next step involved the filtration of the supernatant including the extracted analytes by sequential filtration through a 0.45 µm and 0.22 µm cellulose acetate filters (Labsolute, TH. Geyer, Renningen, Germany) to remove any particulate matter. The cellulose acetate membrane was selected to prevent the retention of lipophilic anthelmintic compounds within the membrane. Despite the intricate composition of the matrix, solid-phase extraction (SPE) cleaning was deemed unnecessary and was therefore omitted. The extracts were frozen at −70 degrees until mass spectrometry on the following day.

An isotope-labeled internal standard was used to account for minor differences in handling and the matrix effect. Since isotope-labeled standards are not commercially available for all four anthelmintics, this study focused on demonstrating the proof of concept for fenbendazole-d₃, as it is readily available. However, for the analysis of real samples, it would be advisable to use isotope-labeled standards for each analyte, if available, to achieve the best possible results.

### Mass spectrometry and data analysis

Mass spectrometry analysis of anthelmintics content in horse manure was performed by high-performance liquid chromatography electrospray tandem mass spectrometry (HPLC-ESI-MS/MS) using a 1290 Infinity II HPLC system (Agilent, Waldbronn, Germany) coupled to a 6500++ QTRAP® mass spectrometer (SCIEX, MA, USA). The 6500++ QTRAP® mass spectrometer was equipped with a TurboV electrospray ion source and was operated in positive electrospray ionization mode set on low mass. Anthelmintics were detected using scheduled multiple reaction monitoring (scheduled MRM). Ammonium formate was included in the tuning solution as well as in the HPLC solvents and instrument parameters were optimized. MRM transitions, energies [V] and ion ratios are shown in Table 1. The dwell time for each MRM transition was max. 250 ms. The following general mass spectrometry settings were established: electrospray voltage 5500 V, curtain gas 50 (arbitrary units), Ion Source One and Two 40 and 40 (arbitrary units) and probe temperature 290 °C.

**Table 1:** MRM conditions and ion ratios.

Q1	Q3	RT [min]	Compound	DP	EP	CE	CXP	Ion Ratio
207.1	150	4.6	Pyrantel_1	70	10	40	15	0.67 ± 3%
207.1	136	4.6	Pyrantel_2	40	10	40	15	(0.40 - 0.94)
300.1	267.9	9.2	Fenbendazole_1	40	10	30	15	0.41 ± 9%
300.1	158.9	9.2	Fenbendazole_2	60	10	50	15	(0.25 - 0.57)
303.1	268.1	9.1	Fenbendazole-d3_1	40	10	30	20	0.34 ± 4%
303.1	158.9	9.1	Fenbendazole-d3_2	50	10	50	20	(0.21 - 0.48)
640.3	528.3	12.2	Moxidectin_1	40	10	15	30	0.61 ± 6%
640.3	498.3	12.2	Moxidectin_2	40	10	15	25	(0.36 - 0.85)
892.5	307.2	12.3	Ivermectin_NH4_1	30	10	35	20	0.67 ± 21%
892.5	551.3	12.3	Ivermectin_NH4_2	35	10	30	20	(0.40 - 0.93)

HPLC separation was performed on a ZORBAX Eclipse Plus C18, 2.1 × 50 mm column (particle size 1.8 µm, pore size 95 Å, Agilent, Waldbronn, Germany) at a flow rate of max 300 µL/min and a column temperature of 50 °C. A lower flow rate of 100 µL/min was employed within the first two minutes of the HPLC gradient to achieve separation of the two pyrantel signals. The mobile phase consisted of 2% methanol, 5 mM ammonium formate and 0.1% formic acid (FA) (solvent A) and 99% methanol, 5 mM ammonium formate and 0.1% FA (solvent B). The following gradient conditions were used (Table 2). Data acquisition and quantification were performed using SCIEX OS 3.3.1.43 software (SCIEX, MA, USA).

**Table 2:** Liquid chromatography conditions.

Time (min)	A (%)	B (%)	Flow (mL/min)
0	100	0	0.1
2.20	100	0	0.1
2.21	100	0	0.3
5.5	50	50	0.3
7.5	45	55	0.3
10.5	25	75	0.3
11	5	95	0.3
11.5	0	100	0.3
16	0	100	0.3
16.1	100	0	0.3
19.8	100	0	0.1
19.9	100	0	0.1

### Matrix effect

The matrix effect of manure was determined by diluting 10 and 50 ng/mL of the four different anthelmintics in quintuplicate into solvent (37.5% H_2_O, 62.5% acetonitrile, 5 mM ammonium formate) as well as into extract from untreated manure. The matrix effect was calculated using the following formula:

$$\frac{Area\;of\;matrix\;peak-Area\;of\;solvent\;peak\;}{Area\;of\;solvent\;peak}*100\;\%$$

## Method validation

### Extraction procedure

The objective of these experiments was to develop a rapid and straightforward method for the direct extraction of various anthelmintics commonly used in veterinary treatment from horse manure and to quantify them in a single HPLC-MS/MS analysis. Initial trials employing various water-to-acetonitrile ratios yielded linear calibration curves for pyrantel and fenbendazole. However, this was not the case for ivermectin and moxidectin. The latter two presented a significant challenge with regard to mass spectrometry tuning. Ivermectin exhibited an optimal response in its ammonium ion adduct form, highlighting the importance of ammonium ion in maintaining the compound's stability [1] made similar observations when analyzing ivermectin in dog plasma. Although the signal intensity for moxidectin in the protonated [M+H]^+^ form was higher than that of the ammonium ion adduct., the ammonium ion adduct was shown to be essential for the linearity of the calibration curve. Therefore, 5 mM ammonium formate was incorporated into the extraction solvent. This resulted in satisfactory signal intensities for all four analytes when MRM detection was employed.

Additionally, it was determined that adjusting the collision and curtain gases was of significant importance, especially when using the QTrap 6500+ system. Consequently, the automatic compound optimization procedure was employed to achieve the optimal performance of the mixture of all four compounds. The resulting values are as follows: electrospray voltage 5500 V, curtain gas 50 (arbitrary units), Ion Source One and Two 40 and 40 (arbitrary units) and probe temperature 290 °C. Furthermore, it became evident that the injecting low volumes onto the column was of paramount importance. Our findings indicated that a maximum injection volume of 2 µL generated narrow and high-intensity signals with low background. Despite the complexity of the matrix, solid-phase extraction (SPE) cleaning was deemed unnecessary and was therefore omitted. The detailed extraction procedure is outlined in the Methods section.

The stability of anthelmintics during drying is critical for sample preparation. Freeze-drying the manure would be a gentle method for drying the samples, but such equipment is not available in every laboratory. Therefore, experiments were conducted to investigate the stability of four anthelmintics when dried at 100 °C in an incubator. Manure samples were spiked in triplicate before and after drying, then extracted according to the described protocol. Despite the high drying temperature, the results showed that all four substances were remarkably stable (data not shown). The effective temperature in the manure was likely significantly lower than the ambient temperature of 100 °C.

### Matrix effect and ion ratio

In mass spectrometry, ionization efficiency is significantly affected by the matrix, or the composition of the extract. Due to the inherent complexity of manure matrices, we conducted a comparative analysis of the matrix effect in conjunction with a pure solvent mixture. While the influence of the matrix on the detection of pyrantel pamoate was minor with values between 4.8 and 10.4%, the effect on the other anthelmintics ranged from 19.7 to 23.3% for ivermectin and up to 58.9% for moxidectin and fenbendazole (see table S1 in supplement). Accordingly, all calibration curves were conducted in the presence of manure extract to account for the matrix effect. However, equine manure is a very difficult matrix that is strongly influenced by the horse's metabolism, age, and diet. If available, isotope-labeled internal standards should be used for all analytes to minimize the influence of differences in manure composition between real samples and the manure extract used for calibration.

In MRM experiments, the ratio between the transitions of each analyte is proof for analyses of the correct compound. For each analyte, the ion ratio was determined as the ratio of the qualifier (MRM transition 2) to the quantifier (MRM transition 1) using the internal calculation of the Sciex OS software (version 3.3.1.43). The numbers in Table 1 were calculated from the mean values of the calibration curves from three independent runs. The standard deviation ranged from 3% to 21%, with ivermectin exhibiting the greatest variability in ion ratio. However, the accepted range in difficult matrices is up to ±40% [10]. The limit values are also provided.

### Calibration curves

We focused on a range of 10 ng to 10 µg of anthelmintics per gram of dry manure weight to evaluate the efficiency of our methodology. This range corresponds to a concentration of approximately 1.2 ng to 1.2 µg/mL concentration in the extracts. These lower concentrations were selected for study, because it has been established that ivermectin and moxidectin can are present in feces for up to 30 days after treatment [8]. Ivermectin, in particular, has been demonstrated to exert an effect on non-target organisms at concentrations that are exceptionally low. For example, the EC50 for the small planktonic crustacean *Daphnia magna* (*D. magna*) is 5.7 ng/L for ivermectin and 12 µg/L for fenbendazole [3]. Moxidectin showed a decrease in germination efficiency and an increase in germination time for the seeds of three prevalent temperate grassland species (*Centaurea jacea, Galium verum, Plantago lanceolata*) when directly applied at concentrations ranging from 10 μg/L to 10 mg/L [4].

All calibration curves were performed in the background of horse manure extract and were found to be linear within the range of 1 ng/mL (lower limit of quantification, LLOQ, signal-to-noise ratio (S/N) at least 10) to approximately 1.5 µg/mL. The lower level of detection (LLOD, S/*N *> 3) was determined to be 0.1 ng/mL for ivermectin, 0.2 ng/mL for moxidectin, 0.3 ng/mL for pyrantel and 1 ng/mL for fenbendazole, respectively. For a detailed discussion on the limitations of pyrantel and fenbendazole detection, please refer to limitations section.

The upper quantification limit (ULOQ) for pyrantel and fenbendazole was determined to be just below 1.5 µg, while for ivermectin and moxidectin, the ULOQ was slightly above 1.5 µg/mL. The ion response of these two compounds was found to be notably lower than that of the other two anthelmintics, consistent with previous publications on the subject [11]. Data analysis was performed using a regression model with a slope of 1/x. The resulting R² values exceeded 0.998 in all cases. The LLOQ values were assigned an accuracy of ± 20%, while all other values were assigned an accuracy of ± 15%, as recommended in the guidelines of the European Medicines Agency [12] and [13]. Values corresponding to these criteria were included in the calibration curves resulting in at least six calibration points. The underlying data are presented in Table 3.

**Table 3:** Calibration curves for all four anthelmintics. All compounds were analyzed in triplicate, and the mean value is reported. The standard deviation (StDv) was below 5% in all cases, except for the lowest concentration of 1 ng/mL.

Concentration in ng/ml	Pyrantel			Fenbendzol			Moxidectin			Ivermectin		
	Average	StDv	StDv in %	Average	StDv	StDv in %	Average	StDv	StDv in %	Average	StDv	StDv in %
	*n *= 3			*n *= 3			*n *= 3			*n *= 3		
1	0.9	0.2	19.8	0.9	0.0	0.6	1.0	0.2	22.4	0.47	0.1	26.0
10	9.9	0.1	0.9	9.6	0.5	5.0	10.4	0.8	7	10.4	0.5	4.6
100	101	6.7	6.7	96	3.2	3.4	101	4.8	4.8	105	5.3	5.1
500	494	21.2	4.3	477	15.4	3.3	488	19.8	4.1	493	8.4	1.7
1000	1025	38.9	3.8	1027	45.6	4.4	1029	17.7	1.7	1052	19.8	1.9
1200	1192	16.8	1.4	1208	17.6	1.5	1173	24.5	2.1	1166	14.1	1.2
1500	1487	25.6	1.7	1493	15.5	1.0	1509	18.9	1.3	1502	47.0	3.1

### Extraction of anthelmintics from horse manure

The extraction method developed in this study was applied to real samples, i.e., the manure of horses that had received a single dose of either pyrantel or ivermectin two days earlier. In both cases, the respective substances could be extracted, yet none of the non-administered anthelmintics (fenbendazole, moxidectin) were detected, thereby demonstrating the efficacy of the method. However, the concentrations of pyrantel and ivermectin detected in real samples were very high, up to 45 µm/g of dried manure. Since the goal of this study was to develop a method for extracting and analyzing very low concentrations of these four anthelmintics, we focused on samples spiked with defined amounts ranging from 10 ng/g to 10 µg/g of dried manure.

**Fig. 1:**
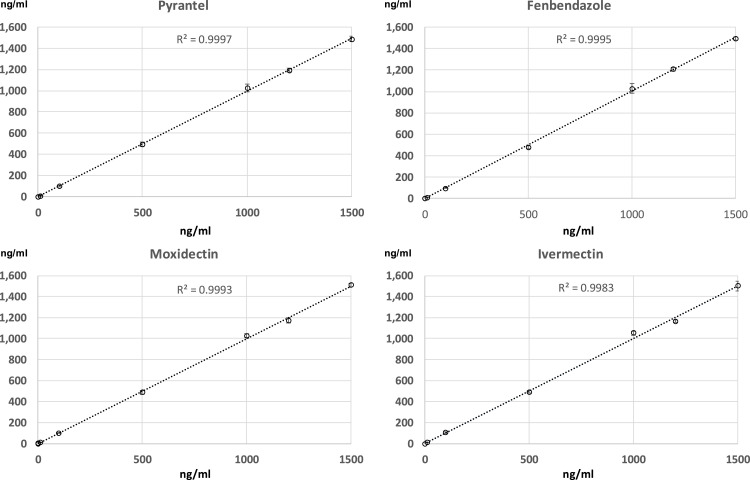
Calibration curves of all four anthelmintics. A regression of 1/x was used to generate calibration curves, and the resulting R² values are listed. All four compounds show linearity within the range of 1 ng/mL and approximately 1.5 µg/mL.

**Fig. 2:**
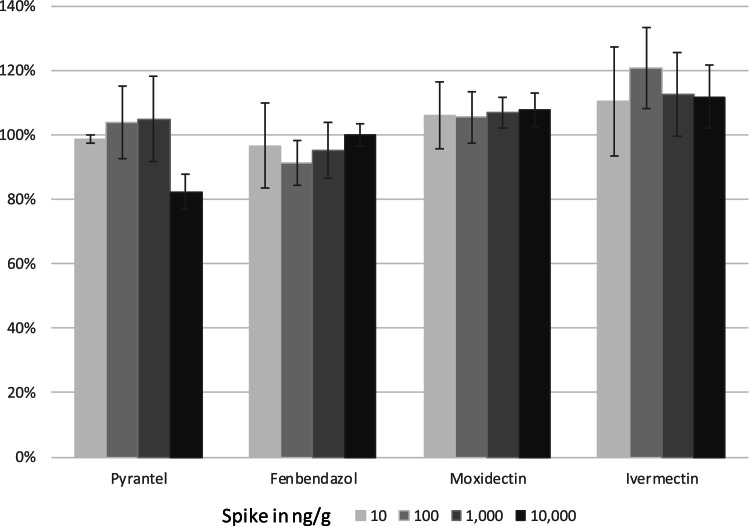
Extraction efficiency of individual anthelmintics in percent. The figure illustrates the extraction efficiency of the four anthelmintics. In the majority of cases, the efficiency for all four compounds is well above 90%, with a standard deviation below 15%.

To assess the efficiency of the novel extraction method, dried horse manure was spiked with anthelmintics at varying concentrations: 10 ng, 100 ng, 1 μg, and 10 µg of each compound were dissolved in 300 μl of DMSO with ethanol with 5 mM ammonium formate and then mixed with 1 g of dried manure. Fenbendazole-d3 (500 μL of 4 μg/mL solution) was added as an ISTD (internal standard). The compounds were extracted and subsequently analyzed using HPLC–MS/MS. The results of these analyses are presented in Table 3 and illustrated in Fig. 1. Spiking experiments were conducted in three separate runs that had been prepared by two different individuals on three different days to assess the inter-day as well as handling variance. The extracts were then analyzed by mass spectrometry on the day of preparation. Calibration curves and dilutions were prepared separately for each extraction using the spiking solutions diluted in the manure extract (Fig. 2) (Table 4, 5).

**Table 4:** Extraction efficiency. Summary of the extraction efficiency of each anthelmintic, expressed as a percentage. The mean value was calculated from three independent trials. StDv denotes standard deviation, and n represents the number of trials. Spike is indicative of the amount of known concentration of the respective anthelmintic.

Spike [ng/g]	Pyrantel		Fenbendazole		Moxidectin		Ivermectin	
	*n *= 3		*n *= 3		*n *= 3		*n *= 3	
	Average	StDv	Average	StDv	Average	StDv	Average	StDv
10	98.7%	1.3%	96.7%	13.3%	106.0%	10.5%	110.2%	16.9%
100	103.8%	11.4%	91.3%	7.0%	105.4%	8.0%	120.8%	12.6%
1000	104.9%	13.2%	95.3%	8.7%	106.9%	4.8%	112.6%	13.1%
10,000	82.3%	5.4%	99.8%	3.4%	107.7%	5.3%	111.8%	9.8%

**Table 5:** Spiking solutions diluted into the matrix in the same ratio as they would appear after extraction. The mean value was calculated from three independent trials. StDv is an abbreviation for standard deviation, while n denotes the number of trials. Diff is an abbreviation for difference.

Dilutions [ng/ml]	Pyrantel			Fenbendazole			Moxidectin			Ivermectin		
	*n *= 9			*n *= 9			*n *= 9			*n *= 9		
	Average	StDv	Diff	Average	StDv	Diff	Average	StDv	Diff	Average	StDv	Diff
1.176	1.3	6.2%	14.8%	1.1	4.7%	−3.2%	1.1	10.6%	−9.7%	1.2	15.0%	3.3%
11.76	14.2	7.8%	20.9%	10.9	5.8%	−7.0%	11.8	7.2%	0.0%	13.9	10.0%	17.8%

The extraction efficiency for pyrantel ranged between 82.3% and 104.9%, for fenbendazole between 91.3% and 99.8%, for moxidectin between 105.4% and 107.7%, and for ivermectin between 110.2% and 120.8% with standard deviations in all but one case below 15%. These findings indicate that our recently developed extraction method is highly effective for all four compounds, despite their diverse chemical compositions. To assess the intraday variance, a set of extracts was injected three times, and the resulting concentrations were compared with the expected values. The standard deviation of the three runs was found to be below 10%, with 13 out of 16 values remaining below 5%. (See supplement Table S2)

To assess the outcomes of the recovery tests, dilution experiments were conducted in which the spiking solutions were directly diluted into the manure extract in the same ratio as it would appear after extraction. Three dilutions were made per day of measurement. The results of the dilution tests are consistent with the anticipated range of 85 to 115%, aligning with the values obtained from the extraction process. The efficacy of the extraction method is therefore confirmed.

Spike samples were stored at −70 °C and analyzed again after eleven weeks (data not shown). While moxidectin remained stable, the other substances decayed slightly. Ivermectin decreased to 83.6%, and both fenbendazole variants decreased to approximately 70%. The greatest loss was observed with pyrantel, which was reduced to only 17.5%. Therefore, we strongly recommend analyzing the manure extracts immediately after preparation and preparing calibration samples freshly each time. In this study, all three extraction runs were performed on consecutive days and analyzed the following day. Standard solutions were not stored for more than four weeks, even when stored at −70 °C.

## Limitations

It is important to note that in the experimental setup, pyrantel showed two distinct peaks with the same *m/z* ratio and fragmentation pattern. This was determined by analyzing the standard compound on a high-resolution Q-Exactive Plus mass spectrometer. This phenomenon has been reported in previous literature and depends on the column and HPLC gradient used [14]. Furthermore, an interfering compound was observed in the second peak at RT = 4.6 min, resulting in the lower limit of quantification (LLOQ) being determined to be 1 ng/mL and the level of detection (LOD) being 0.3 ng/mL when the first peak appeared at RT = 4.3 min and S/N was >3. The analysis revealed an increase in both peaks in response to elevated concentrations of pyrantel. However, the ratio of the two peaks was inconsistent, even when standard dilutions were employed. Consequently, the peak areas of the two peaks were combined for quantitation. One possible explanation is that this second peak and interfering compound could be due to the pyrantel embonate impurity A (Compound CID: 6914,280), which is a *cis* isomer of pyrantel.

Fenbendazole was observed to adhere to the column, resulting in carry-over. Despite implementing a 4.5-minute cleaning step with a high concentration of organic solvent, a four-minute reequilibration time in each run, cleaning the column after each series, and washing it backwards, the carry-over persisted. Therefore, after runs with high fenbendazole concentrations, three blank runs were performed to reduce the carry-over signal as suggested in the guidelines of the European Medicines Agency [12]. This reduced the carry-over signal to <20% of the LLOQ signal and 5% of the internal standard signal as suggested in these guidelines. In addition, the 0 ng/mL point was established as the baseline, meaning its value was substracted from each calibration point and sample. This process facilitated the accounting for the carry-over signal. The LOD and the LOQ were both defined at 1 ng/mL due to the carryover effect. The upper limit of quantification (ULOQ) ranges from 1.2 µg/mL to 1.5 µg/mL, with the upper value approaching the upper detection limit of 1.0 E8 of the QTRAP 6500+.

Due to its fluffy consistency, ivermectin must be handled very carefully when weighing in while preparing the standard solution. Ivermectin is characterized by its tendency to disseminate and disperse, which causes background signals at the very low concentrations.

## Ethics statements

None.

## Credit author statement

**Katharina Holzer:** Conceptualization, Methodology, Extraction, Illustration, Writing- Original draft preparation. **Ute Bertsche:** Conceptualization, Methodology, HPLC-MS/MS analysis, Validity tests, Data curation, Illustration, Writing- Original draft preparation, Editing. **Madeline Meyer:** Material, Writing-Reviewing, **Thorben Schilling:** Conceptualization, Writing- Reviewing, **Jens Pfannstiel:** Validation, Writing- Reviewing, Supervision**. Ludwig Hoelzle:** Conceptualization, Writing- Reviewing, Supervision.

## Declaration of generative AI and AI-assisted technologies in the writing process

During the preparation of this work the authors used DeepL Write to enhance the quality of writing and ensure the correct use of grammatical conventions. After using this tool, the authors reviewed and edited the content as needed and take full responsibility for the content of the published article.

## Declaration of interests

The authors declare that they have no known competing financial interests or personal relationships that could have appeared to influence the work reported in this paper.

## Data Availability

Data will be made available on request.

## Appendix Supplementary materials

Image, application 1
