# Supplementary material for: Simple and efficient HPLC-MS/MS method for simultaneous extraction and quantification of anthelmintics in equine manure
Source: MethodsX. 2026 Jul 12;17:104042. doi: 10.1016/j.mex.2026.104042 (PMC13377484; doi:10.1016/j.mex.2026.104042)
Supplement: Supplementary file 1 [file mmc1.docx]

**Supplementary material *and/or* additional information [OPTIONAL]**

**Supplement**

**Table S1: Matrix effect**

The matrix effect for manure in comparison to solvent was measured and demonstrated to be significant for three of the four compounds, as well as the internal standard. The values obtained for this study ranged from 20 to nearly 60%.

**Fig. S1: Chromatograms of the four anthelmintics at lower concentrations**

The figure illustrates the MRM chromatograms of the four anthelmintics at low concentrations (matrix, 0 ng spike, 1 ng spike, and 10 ng spike). Pyrantel shows two peaks, the second peak is probably due to pyrantel embonate. Fenbendazole shows carry-over effects producing signals in both the matrix and the 0 ng spike samples.

**Table S2: Intraday reproducibility and instrument performance**

To assess the performance of the instrument, a set of extracts was injected three times on the same day. The standard deviation was found to be less than the requested 10%, and in 13 out of 16 cases, it was even found to be less than 5%.
